# Haptic Technology: Exploring Its Underexplored Clinical Applications—A Systematic Review

**DOI:** 10.3390/biomedicines12122802

**Published:** 2024-12-10

**Authors:** Kevin Pacheco-Barrios, Jorge Ortega-Márquez, Felipe Fregni

**Affiliations:** 1 Neuromodulation Center and Center for Clinical Research Learning, Spaulding Rehabilitation Hospital and Massachusetts General Hospital, Harvard Medical School, Boston, MA 02115, USA; kpachecobarrios@mgh.harvard.edu (K.P.-B.); jorge_ortegamarquez@hms.harvard.edu (J.O.-M.); 2 Unidad de Investigación para la Generación y Síntesis de Evidencias en Salud, Vicerrectorado de Investigación, Universidad San Ignacio de Loyola, Lima 15023, Peru

**Keywords:** haptics, haptic technology, haptic training, multisensory training systems, medical devices, rehabilitation, mental health, cognition, medical innovation

## Abstract

Background/Objectives: Haptic technology has transformed interactions between humans and both tangible and virtual environments. Despite its widespread adoption across various industries, the potential therapeutic applications of this technology have yet to be fully explored. Methods: A systematic review of randomized controlled trials (RCTs) and randomized crossover trials was conducted, utilizing databases such as PubMed, Embase, Cochrane Library, and Web of Science. This review included studies reporting clinical applications of haptic technology in rehabilitation, cognition, wellness, and mental health among adult subjects. Results: This systematic review included 34 studies, of which 20 focused on clinical outcomes and 14 on learning clinical skills. The results showed that haptic devices, both robotic and non-robotic, enhance sensorimotor performance and motor function in rehabilitation settings, especially in post-stroke recovery, with reported effect sizes ranging from 0.2 to 0.7. The majority of the haptic technologies reported were integrated into robotic systems (40%). Haptic devices were also reported to improve clinical skills training by providing tactile feedback that enhances procedural performance and trainee self-efficacy. In fact, surgical simulations accounted for 79% of all the modalities used for medical training. Conclusions: This review underscores the potential yet underexplored applications of haptic technology in healthcare, including medical education, rehabilitation, cognition, and mental health. The key limitations of this review include heterogeneity across studies, small sample sizes, and a scarcity of comprehensive, long-term investigations. Therefore, future research should aim to validate these findings further and expand the applications of haptic technology to maximize its utility in the healthcare industry and clinical practice.

## 1. Introduction

Haptics is a word derived from the Greek root “haptikos” and refers to the sense of touch related to the perception and manipulation of objects in both natural and synthetic environments [1]. Haptic systems were first introduced in the 1960s and 1970s in the aircraft industry, providing pilots with pre-stall warnings through natural control vibrations, and later expanded into the communication industry with the invention of the first tactile man–machine communication system [2]. During the last decades, haptic technology has undergone significant advancements, and its applications have shown great promise in transforming human interactions with both real and virtual environments [3]. Despite this evidence, further investigation of haptic devices is necessary to enhance the understanding of human interactions with the external world through this technology and explore possible applications.

Haptic systems are primarily categorized into two essential types: kinesthetic, which involves positions and forces related to muscles, tendons, and joints, and cutaneous, which pertains to tactile sensations related to the skin [4]. Each haptic system fundamentally consists of two essential components: the human component, which includes nerve receptors necessary for sensing, and the machine component, which provides the forces needed to simulate the contact and interaction with tangible or intangible objects. These components are usually integrated with closed-loop or feedback systems, sensors, processors, and actuators [5]. The principles underlying these technologies have promoted the development of broadly three types of haptic devices: (1) graspable devices, such as human-controlled teleoperation robotics in surgical, military, and manufacturing machinery [6,7,8]; (2) touchable devices, such as those used for shape identification [9,10]; and (3) wearable devices, including fingertip, glove-based, or exoskeleton devices [11]. These technologies have been integrated into multiple sectors, including the use of tools that allow the visually impaired population to independently create virtual graphs and images [12]; the automotive industry with control visual–haptic feedback and interactive touchscreens [13]; and virtual education, which incorporates sensory data and feedback for kinesthetic or auditory learners [14]. Interestingly, haptics does not function as an isolated system; it can integrate and combine information with other senses and systems, such as visual, auditory, proprioceptive, and vestibular systems, for different purposes [15,16,17,18]. Examples include the incorporation of visual and tactile stimuli through virtual and augmented visual reality (VR/AR) with haptic wearables in STEM education [19] and the creation of the next generation of surgical robotics integrating multisensory surgical support systems, including tactile information together with visual and audio feedback [20]. While the diverse discovery of haptic devices marked the beginning of discovering their potential, their various application areas remain underexplored.

Healthcare stands at an initial yet promising stage in the development of haptic technology. Firstly, haptics has introduced new teaching methods in medical and dental education with the development of devices designed to improve clinical skill acquisition, such as shadow hands, stitching simulators, and periodontal or dental implant simulators [21,22]. Haptic technology has shown promising results in improving clinical practice. Examples include the performance of medical interventions by using the nature of haptic information and its perception in remote surgery [23,24] and interventional radiology procedures [25,26]. In addition, haptic devices, when combined with technologies such as virtual reality and artificial intelligence, have been shown to facilitate interactions with medical devices among patients and healthcare providers. As an illustration, during the COVID-19 pandemic, assistive technologies based on haptics, robotics, and simulators provided a viable alternative to reduce physical contact in medical institutes, thereby reducing the risk of contagion and patient complications [27]. Initial evidence has also suggested clinical applications, such as in the field of rehabilitation, where haptic technology has been reported to be a tool in the recovery of stroke [18,28], spinal cord injury [29], Alzheimer’s disease [30], and mental health conditions [31]. Despite these advancements, the clinical application of haptics remains underexplored.

To the best of our knowledge, there has not yet been a systematic and comprehensive synthesis of the current literature that allows for a high-quality evaluation of haptic technology’s clinical applications. As a result, this systematic review aims to explore the current literature concerning the clinical applications of haptic technology in areas such as rehabilitation, cognition, wellness, and mental health. We also intend to provide a comprehensive list of devices that are available for clinical applications. Additionally, we aim to identify specific populations, including patients, physicians, and educators, who could benefit most from this technology. By examining these aspects, our study seeks to deliver a novel, integrative, and high-quality assessment of the clinical applications of haptic devices.

## 2. Materials and Methods

This systematic review was registered with PROSPERO (CRD603031) and conducted based on the Preferred Reporting Items for Systematic Reviews and Meta-Analyses (PRISMA) guidelines. By adhering to the PRISMA guidelines, we followed a globally recognized framework that dictates a structured process for conducting and reporting systematic reviews. This approach helps in maintaining consistency, allowing for a comprehensive synthesis of the evidence and facilitating the comparability of our review with other studies in the field. In addition, the use of PRISMA guidelines specifically aids in the detailed and transparent reporting of each step, from the literature search to study selection and synthesis, ensuring that all relevant studies are considered and appropriately integrated [32].

### 2.1. Search Strategy

A search strategy was established based on randomized controlled trials (RCTs) and randomized crossover trials published up to July 2024 in PubMed, Embase, Cochrane Library, and Web of Science. The following terms were used as keywords for the search strategy: Haptic Technology (haptic interfaces, haptic, vision entoptic); Cognition (cognitive, attention, memory); and Mental Health (brain health, mood disorders, depression, mood disorders, stress). The search strategies are available in Supplementary Table S1. In addition, we conducted a systematic search in the Bloomberg and Crunchbase databases to identify current trends in haptic technology development across markets and to analyze companies primarily focused on developing haptic devices. The following keywords were used: “haptic technology”, “tactile feedback”, “force feedback”, “vibrotactile”, and “haptic interface”.

### 2.2. Study Eligibility and Selection

**Figure 1:**
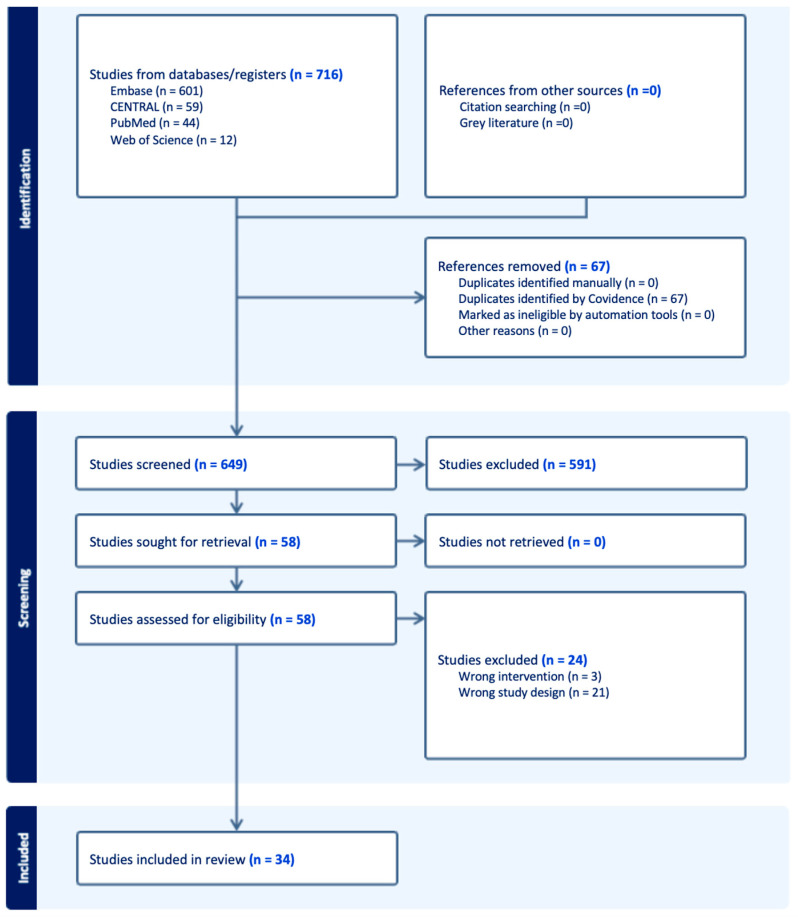
PRISMA flow diagram showing search results.

The search strategy was limited to studies written in English. Inclusion criteria: (1) RCTs; (2) crossover randomized trials; (3) studies using haptic technology/devices and reporting clinical outcomes; (4) adult patients (>18 years old). Exclusion criteria: (1) Reviews; (2) meta-analyses; (3) case reports; (4) conference abstracts not published as full manuscripts; (5) study protocols; (6) population < 18 years old and animals; (7) studies in which outcome assessments were not provided or could not be calculated from initial or supplementary data. Eligibility assessment was performed following the flowchart sequence (Figure 1). First, two independent reviewers (J.O.-M.and K.P.-B.) performed title and abstract screening. Conflicts were solved by a third independent reviewer (F.F.). Then, two authors (J.O.-M.and K.P.-B.) assessed the full texts of the remaining articles to verify inclusion/exclusion criteria. Again, conflicts were resolved by a third independent reviewer (F.F.).

### 2.3. Data Collection and Quality Assessment

When reviewing studies, we collected the following variables from each study: (1) title, (2) author, (3) country, (4) publication year, (5) study design, (6) sample size, (7) mean age (and standard deviation) and gender of participants, (8) type of condition or disorder, (9) type of haptic technology/intervention, (10) clinical outcome measured, (11) technique or metric used to measure clinical outcomes. For the collection of study variables, two independent reviewers (J.O.-Mand K.P.-B.) extracted data separately. Any conflicts were resolved by a third independent reviewer (F.F.) if necessary. This approach minimizes bias and errors by allowing for cross-checking of the extracted data. A quality assessment of the studies included was performed using version 2 of the Cochrane risk-of-bias tool for randomized trials (RoB-2) [33]. To determine the RoB-2 of the eligible articles, two independent reviewers (J.O.-M and K.P.-B.) assessed the quality of each study for selection, detection, performance, attrition, and reporting biases. The following categories were used for evaluating each parameter: low, unclear, and high risk. A third independent reviewer (F.F.) solved conflicts if necessary.

### 2.4. Narrative-Qualitative Data Synthesis

A three-stage narrative synthesis strategy to synthetize the evidence was performed by two reviewers (J.O.-M and K.P.-B.) based on Popay et al.’s 2016 guidelines [34]. Popay et al.’s 2016 guidelines outline a comprehensive framework for narrative synthesis in literature reviews, detailing key elements of the process and offering various methods and tools for data management, manipulation, and synthesis across multiple studies, culminating in the presentation of findings. Based on these guidelines, the key elements and steps considered to synthetize this review were (1) developing a theory on the potential clinical applications of haptic devices; (2) developing a preliminary list of synthesis categories based on different types of clinical applications; (3) presenting the synthesis in summary tables and figures.

## 3. Results

### 3.1. Study Selection

The search strategy yielded a total of 649 records after duplicates were removed. Of these, 591 papers were excluded after title and abstract screening. Of the remaining 58 articles, 24 studies were excluded after full-text screening. Of those excluded, three reported the wrong intervention, and twenty-one had an inadequate study design (Figure 1).

### 3.2. Evidence Summary

In total, 34 studies were included in the analysis, of which 20 were related to clinical outcomes, and 14 were related to clinical skills learning.

(A) Clinical Outcomes.

Of the included studies reporting clinical outcomes, 75% were randomized clinical trials (RCTs), and 25% were crossover designs. The types of populations selected in the trials were healthy adults (45%); adult patients with subacute or chronic stroke (25%), trans-radial amputation (5%), or femoroacetabular impingement (FAI) (5%); adolescents with developmental coordination disorder (DCD) (5%); and children with cerebral palsy (CP) and developmental dyspraxia (DD) (5%), dysgraphia (5%), or manual control difficulties (5%). 

The reported haptic modalities were used in the following anatomical stimulus locations: hand (60%), arm (20%), fingers (10%), forearm (5%), and chest (5%). The majority of these modalities were integrated into robotic systems (40%), for instance, the application of a robotic hand–forearm to stimulate the motor system in subjects with acute stroke [35], the employment of a robotic hand interface for enhancing motor recovery in chronic stroke when combined with therapist-assisted arm mobilization [36], or the utilization of a robotic arm to separate the effects of action observation on motor learning from the effects of haptic guidance in healthy adults [37]. Additionally, the implementation of a robotic arm integrated with a virtual game-like and audio-haptic stimulating motivation system was carried out in healthy adults [38]. Training/condition setting programs (35%) were also reported as haptic modalities, such as the haptic perception training program (HPT) employed to stimulate the sensorimotor system in adolescents with developmental coordination disorder (DCD) [39]. Other non-robotic interventions (25%) were noted, for example, the immersion virtual gaming program used in children with cerebral palsy and developmental dyspraxia to improve motor and kinesiological performance [40].

The clinical outcomes measured in studies using haptic technologies were primarily focused on motor recovery (75%), mainly in patients with stroke, using robot-assisted haptic technology [35,36,41,42,43]. Other populations also reported motor performance as a clinical outcome, including children with cerebral palsy using a haptic perception training program [40], adolescents with developmental coordination disorder (DCD) using an immersion virtual gaming program [39], and trans-radial amputees patients or diagnosed with FAI [44,45]. Additional clinical outcomes reported in the studies were sensorimotor performance (5%) and balance (postural sway) (5%) in elderly subjects [46,47], as well as motivation (5%) and interoceptive abilities (5%) in healthy adults [38,48,49,50,51].

Overall, the studies reported a clinically relevant improvement in sensorimotor performance and balance by including haptic modalities as part of robot-integrated systems or independent training programs. The effect sizes ranged from 0.2 to 0.7, and the largest improvement was in motor function after stroke. See Supplementary Table S2 for detailed information about studies reporting clinical outcomes.

(B) Clinical Skills Training.

Among the included studies reporting the use of haptic technology for clinical skills training, 78% were RCTs, and 12% were crossover designs. The populations selected for the studies included specialists (14%), clinical fellows (7%), post-graduate-year residents (50%), and medical or dental students (43%).

The reported haptic technologies included haptic simulators (50%), training (40%), and wearable devices (10%). The haptic simulators were mainly focused on implementing virtual reality or 3D modalities to simulate anatomic graphs and tactile-force sensations within a surgical environment or procedure [52,53,54,55,56,57,58,59,60]. On the other hand, haptic interventions based on training used physical objects or models to simulate the feel and sensations of the surgical procedures [61,62,63,64,65]. One study reported a bracelet as a wearable device with vibrating motor technology to improve tissue-handling skills [62].

The main area where haptic technology was implemented in medical training was surgery (79%), for example, hands-on surgical training (HoST) to enhance urethrovesical anastomosis (UVA) [51] and haptic simulators to improve visual–spatial ability learning, confidence, and knowledge of surgical steps [52,53,54,55]. In addition, wearable devices were used to learn and improve performance in laparoscopic suturing procedures [62]. Other areas in clinical training were also reported, such as anesthesiology (7%), using haptic simulation training for epidural anesthesia skill acquisition [61]; gynecology and obstetrics (7%), using a transvaginal high-fidelity simulator to produce adequate virtual transvaginal ultrasound images in inexperienced trainees 56; and dental skills (7%), implementing a haptic virtual reality (VR) training system to improve performance in skill acquisition and retention [59].

Overall, the studies reported a significant improvement in the procedure performance and higher self-efficacy of the participant during the clinical execution of the task, especially in surgical simulators. See Supplementary Table S3 for detailed information about studies reporting haptic technology for clinical skills training.

### 3.3. Market Trends in Haptic Technology Development

In total, ten leading companies were identified as major contributors to the haptic technology industry focused on clinical applications, as detailed in Table 1.

### 3.4. Risk of Bias

Most of the included studies were evaluated as having low risk for all outcomes, except for eight studies, which showed some concerns [43,51,53,54,55,58,62,66]. See Supplementary Figure S1 for detailed judgments for each risk-of-bias domain criterion.

## 4. Discussion

Our study delved into the current literature on the clinical applications of haptic technology, focusing on rehabilitation, clinical skills training, wellness, and mental health. Our analysis included 34 studies, with 20 investigating clinical outcomes and 14 exploring clinical skills learning. The studies demonstrated the potential of haptic technology to enhance sensorimotor performance, particularly in post-stroke rehabilitation, and to improve clinical skills training, particularly in surgical simulations. Despite the promising results, the exploration of haptic technology remains in its infancy, with numerous applications yet to be fully explored.

### 4.1. Haptic Devices in Rehabilitation

Haptic technology, particularly in the form of robot-integrated systems and non-robotic devices, has shown considerable promise in rehabilitation settings [67]. The studies included in this review predominantly focused on motor activity and performance, with 75% of the studies reporting clinically significant improvements. This suggests that haptic devices can effectively provide real-time feedback to users, facilitating motor learning and enhancing sensorimotor performance [68]. For example, in the post-stroke rehabilitation population, haptic feedback was found to improve motor function, with effect sizes ranging from 0.35 to 0.87. These findings underscore the potential of haptic technology to serve as a valuable tool in the rehabilitation of various conditions, including stroke, cerebral palsy, and developmental dyspraxia, where sensorimotor function is compromised [69].

The success of haptic devices in these settings can be attributed to their ability to provide users with a sense of touch or force feedback that mimics real-life interactions [69]. This sensory input can help users to better understand and control their movements, leading to improved outcomes in rehabilitation [70]. Moreover, the integration of haptic feedback into training programs may enhance motivation and engagement, further contributing to the effectiveness of these interventions [71]. However, the diversity of the haptic modalities and the anatomical locations targeted in these studies suggests that more research is needed to determine the most effective configurations and applications of haptic technology in different patient populations.

Haptic perception is crucial for motor learning, as it provides essential feedback that guides the refinement and adaptation of motor skills [72]. The sense of touch, including the perception of pressure, texture, and force, allows individuals to interact with their environment in a precise and controlled manner [73]. During motor learning, haptic feedback offers real-time information about the success or failure of movements, enabling the nervous system to make necessary adjustments to improve performance [73]. This tactile feedback is especially important in complex motor tasks that require fine motor control, such as grasping objects, manipulating tools, or performing surgical procedures. By continuously processing haptic input, the brain can update motor plans, enhance muscle coordination, and solidify motor memory through practice [73]. The integration of haptic cues with visual and auditory information further enhances the accuracy and efficiency of motor learning, making it a multisensory experience that is vital for acquiring and refining new skills. In essence, haptic perception acts as a critical feedback loop that supports the development of precise and effective motor behaviors, ultimately leading to improved motor performance and skill retention [74]. Future device development should focus on integrating haptic training with other sensorial modalities according to the patient population and individual deficiencies. This will enhance the feedback loop and optimize the sensorimotor rehabilitation process.

### 4.2. Haptic Devices in Clinical Education and Simulation

Haptic technology has also made significant strides in clinical education, particularly in the training of surgical skills [75]. Among the studies reviewed, 78% were RCTs, emphasizing the robustness of the evidence supporting the use of haptic devices in this context. The primary areas of focus were surgery, anesthesiology, gynecology, and dental skills, with the majority of studies utilizing haptic simulators. The findings indicate that haptic technology can enhance procedural performance and self-efficacy among trainees, particularly in surgical simulations, where tactile feedback is crucial [76].

The use of haptic devices in clinical education offers several advantages. First, it allows trainees to practice and refine their skills in a controlled, risk-free environment, reducing the likelihood of errors in real-life clinical settings [77]. Second, haptic feedback can help trainees develop a more nuanced understanding of the tactile aspects of clinical procedures, which are often difficult to convey through traditional training methods [78]. This may be particularly beneficial in fields like surgery, where the sense of touch is integral to performing delicate tasks. The success of haptic devices in this area suggests that they could be further integrated into medical education, potentially revolutionizing the way clinical skills are taught and assessed.

### 4.3. Potential Applications and Research Recommendations

Compared to existing review articles, this paper stands out as a pioneer in providing an integrative overview of the clinical applications of haptic technology. While the current literature has focused primarily on the use of haptic technology in motor rehabilitation and clinical skills training, there are several other applications that have yet to be fully explored. One such area is cognitive improvement, especially in older adults. Haptic devices could potentially be used to enhance cognitive function and learning by providing sensory stimulation that engages the brain pathways [79]. For example, haptic feedback could be incorporated into cognitive training programs to improve attention, memory, and other cognitive skills in older adults [80,81].

Haptic training’s potential to improve cognition can be explained by several plausible mechanisms rooted in the neuroplasticity of the brain and the multisensory integration process [82,83]. One promising application of haptic technologies is their use in enhancing cognitive function and pain modulation. In a previous study, our group demonstrated that somatosensory tasks performed without visual input, similar to the device DOT PAD (DOT Inc., Seul, Republic of Korea), increased pain thresholds in the trained hand while decreasing them in the untrained hand. Additionally, sensory stimulation without visual feedback led to altered cortical excitability, measured by TMS [84]. This suggests that haptic devices may modulate cortical activity and could be used to enhance cognitive function. In fact, haptic feedback engages the somatosensory system, which is intricately linked to cognitive functions such as attention, memory, and spatial awareness [85,86,87]. By providing tactile stimuli, haptic training enhances sensory processing and promotes the integration of sensory information across multiple modalities, thereby strengthening neural connections [88]. This process can lead to improved cognitive performance, particularly in tasks that require fine motor skills, spatial reasoning, or executive function [80]. Moreover, haptic training can stimulate brain regions involved in both sensory and cognitive tasks, such as the parietal and prefrontal cortices, fostering a more coordinated and efficient neural network [89,90]. The repetitive and goal-directed nature of haptic tasks may also facilitate the development of procedural memory and enhance attentional control, further contributing to cognitive improvements [91,92,93]. Through these mechanisms, haptic training not only aids in motor skill acquisition but also offers a promising avenue for cognitive enhancement, particularly in populations with cognitive impairments or those at risk of cognitive decline.

Another promising application is the training of blind or visually impaired individuals. Haptic technology could be used to develop tactile maps or other sensory aids that help these individuals navigate their environment more effectively [94,95]. Additionally, haptic devices could be used in conditions where sensorimotor aspects are disrupted, such as Parkinson’s disease or multiple sclerosis. By providing real-time feedback and sensory input, haptic technology could help individuals with these conditions maintain or improve their motor function and quality of life [85,96,97]. Despite the potential of these applications, they remain underexplored in the current literature. Future research should focus on investigating the feasibility and effectiveness of haptic technology in these and other areas, potentially opening up new avenues for clinical intervention.

The application and implementation of haptic technology require the consideration of three key elements: patients, researchers, and the costs related to its implementation. Firstly, none of the studies reviewed reported moderate or severe adverse effects in patients. Additionally, there were no data indicating any inconvenience associated with the use of haptic devices, underscoring their safe and comfortable use in patient care. This highlights the use of this technology to enhance the usability and interaction of patients with rehabilitation interventions and medical devices. Secondly, from a research perspective, further development of haptic technology could provide new alternatives as a multisensory stimulation tool for various conditions, such as rehabilitation, cognition, wellness, mental health, or visual impairment, as highlighted in this paper. Lastly, regarding cost considerations, while the initial expenses for haptic technology can be high at this stage of development, ongoing advancements promise to create more accessible and cost-effective modalities of this technology.

### 4.4. Limitations

While the findings of this review highlight the potential of haptic technology in various clinical settings, several limitations must be acknowledged. First, the heterogeneity of the studies included in this review makes it difficult to draw definitive conclusions about the effectiveness of haptic technology across different populations and clinical contexts. The studies varied widely in terms of the types of haptic devices used, the populations studied, and the outcomes measured, which may have introduced bias and variability into the findings. Second, the relatively small sample sizes and short durations of many of the studies may limit the generalizability of the results. Larger, long-term studies are needed to confirm the findings of this review and to provide a more comprehensive understanding of the potential benefits and limitations of haptic technology in clinical settings. Finally, the review was limited by the availability of high-quality studies on the topic. While the included studies were generally well conducted, the overall quality of the evidence was moderate, with some studies lacking rigorous methodological designs. Consideration of the additional limitations associated with the assumptions underlying the use of haptic devices is also important. Firstly, although the use of this technology has been well received by patients and providers in terms of usability and safety, significant gaps remain in the understanding of the technology’s interactions with and effects on human sensorimotor systems for therapeutic purposes. These gaps hinder a comprehensive understanding of its human impact, as well as its potential therapeutic effects. Moreover, while reports generally indicate a positive effect of this technology for clinical applications, the existing literature is heterogeneous, with different applications and modalities of this technology. Additionally, despite the absence of major adverse events in the studies analyzed, further testing, validation, and regulatory approval are required to ensure the safety and usability of this technology for clinical applications in human subjects. Therefore, future research should aim to address these limitations by conducting larger, high-quality studies that investigate the effects and use of haptic technology in a broader range of clinical settings and populations.

## 5. Conclusions

In conclusion, this systematic review provides a comprehensive evaluation of the current literature on the clinical applications of haptic technology, yielding several important insights. Firstly, the findings suggest that haptic devices have a significant effect in enhancing sensorimotor performance in rehabilitation settings, especially in post-stroke recovery, and in improving clinical skills training, particularly in surgical simulations. Secondly, based on the information reported in the included studies, haptic technology can be particularly beneficial in post-stroke rehabilitation and in the training of medical professionals, where tactile feedback plays a critical role in skill acquisition and recovery processes. These technologies offer a more interactive and engaging approach to rehabilitation and training, which can lead to better patient outcomes and more skilled healthcare professionals. Thirdly, while the current evidence is promising, the exploration of haptic technology in clinical settings is still in its early stages. Future research should aim to explore a wider range of applications, such as treating cognitive impairments, enhancing mental health, and aiding in the rehabilitation of other neurological conditions with multisensory disruptions, such as Parkinson’s disease, multiple sclerosis, or visual impairment. Research should also explore the integration of haptic technology with other sensory modalities to enhance the multisensory integration processes that are crucial in rehabilitation.

## Figures and Tables

**Table 1:** Leading global companies specializing in haptic technology for clinical applications.

Company	Foundation	Location	Technology Description
Immersion Corporation	1993	USA	Haptic technology for medical simulation and training.
Haption	2001	FR	Real-time haptic interfaces for surgical training and diagnostics.
Synaptics Incorporated	1986	USA	Provides touch, display, and biometrics technology, crucial for medical devices that require sophisticated haptic feedback.
Microchip Technology Inc.	1989	USA	Designs essential components like microcontrollers and integrated circuits for haptic feedback systems in medical devices.
ON Semiconductor Corporation	1999	USA	Develops sensors and controllers for energy-efficient haptic solutions in clinical environments.
Ultraleap Holdings Ltd.	2013	UK	Touchless haptic technology and hand tracking for virtual reality applications in healthcare.
DOT	2014	KR	Develops innovative haptic technology, primarily focused on creating accessible devices for the visually impaired.
Johnson Electric Holdings Limited	1959	HK	Engineers motion subsystems and haptic components for use in various medical devices.
3d Systems Corporation	1986	USA	3D printing and additive manufacturing technologies, including haptic devices for medical modeling.
SMK Corporation	1925	JP	Manufactures electronic components, including those for haptic interfaces used in clinical instruments.

## Supplementary Materials

The following supporting information can be downloaded at: https://www.mdpi.com/article/10.3390/biomedicines12122802/s1, Table S1: Search strategies for the online databases Pubmed (NCBI), Embase, Cochrane; Table S2: Descriptive information of studies related to Clinical Outcomes; Table S3: Descriptive information of studies related to Clinical Skills Training.; Figure S1: Risk of bias of studies included.
