# Supplementary material for: Haptic Technology: Exploring Its Underexplored Clinical Applications—A Systematic Review
Source: Biomedicines. 2024 Dec 10;12(12):2802. doi: 10.3390/biomedicines12122802 (PMC11673350; doi:10.3390/biomedicines12122802)
Supplement: Supplementary file 1 [file biomedicines-12-02802-s001.zip › biomedicines-3341194-supplementary.pdf]

Haptic Technology: Exploring Its Underexplored Clinical Applications — A Systematic Review

Supplementary Material:

Supplementary Table 1. Search strategies for the online databases Pubmed (NCBI), Embase, Cochrane.

**Pubmed (NCBI) search**
("Haptic Interfaces"[Mesh] OR "Haptic Technology"[Mesh] OR haptic[TIAB] OR "Vision, Entoptic"[Mesh]) AND ("Cognition"[Mesh] OR "Attention"[Mesh] OR "Memory"[Mesh] OR "Mood Disorders"[Mesh] OR "Affect"[Mesh] OR "Stress, Psychological"[Mesh] OR "Anxiety"[Mesh] OR "Mental Health"[Mesh] OR "mental health"[TIAB] OR "brain health"[TIAB] OR "depression"[TIAB] OR "cognitive"[TIAB]) AND ("Clinical Trial"[Publication Type])
**Embase search**
('haptic interfaces'/exp OR 'haptic technology'/exp OR haptic:ti,ab OR 'vision, entoptic'/exp) AND ('cognition'/exp OR 'attention'/exp OR 'memory'/exp OR 'mood disorders'/exp OR 'affect'/exp OR 'stress, psychological'/exp OR 'anxiety'/exp OR 'mental health'/exp OR 'mental health':ti,ab OR 'brain health':ti,ab) AND ('clinical trial'/exp OR 'clinical study'/exp)
**Web of Science**
("Haptic Interfaces" OR "Haptic Technology" OR haptic OR "Vision, Entoptic") AND (Cognition OR Attention OR Memory OR "Mood Disorders" OR Affect OR "Stress, Psychological" OR Anxiety OR "Mental Health" OR "mental health" OR "brain health" OR depression OR cognitive) AND ("Clinical Trial")
**Cochrane search**
([mh "Haptic Interfaces"] OR [mh "Haptic Technology"] OR haptic:ti,ab OR [mh "Vision, Entoptic"]) AND ([mh Cognition] OR [mh Attention] OR [mh Memory] OR [mh "Mood Disorders"] OR [mh Affect] OR [mh "Stress, Psychological"] OR [mh Anxiety] OR [mh "Mental Health"] OR "mental health":ti,ab OR "brain health":ti,ab OR depression:ti,ab OR cognitive:ti,ab)

Study ID	D1	D2	D3	D4	D5	Overall
Ranzani 2020	+	+	+	+	+	+
Baur 2018	+	+	+	+	+	+
Chowriappa 2015	+	+	+	+	+	+
Hsu 2021	+	+	+	+	+	+
Lim 2016	+	+	+	+	+	+
Strom 2006	+	+	+	!	+	!
Kantar 2020	+	+	+	+	+	+
Mueller 2013	+	+	+	+	+	+
Dobrushina 2024	+	+	+	+	+	+
Salvador 2024	+	+	+	+	!	!
Rowland 2024	+	+	+	+	+	+
Wuang 2022	+	+	+	+	+	+
Postema 2021	+	+	+	+	+	+
Bortone 2020	+	+	+	+	+	+
Hagelsteen 2019	+	+	!	+	+	!
Childs 2018	+	+	+	+	+	+
Alleblas 2017	+	+	+	+	+	+
Vapenstad 2017	+	+	!	+	+	!
Yoon 2017	+	+	+	+	+	+
Chang 2017	+	!	+	+	+	!
Shire 2016	+	+	+	+	+	+
Brown 2015	+	+	+	+	+	+
Chao 2015	+	+	+	+	+	+
Patel 2014	+	+	+	+	+	+
Ang 2014	+	+	+	+	+	+
Timmermans 2014	+	+	+	+	+	+
Abdollahi 2014	+	+	+	+	+	+
Cameirao 2012	+	!	+	+	+	!
Zadravec 2011	+	+	+	+	!	!
Brokaw 2011	+	+	+	+	+	+
Thompson 2011	+	!	+	+	+	!
Suebnukarn 2010	+	+	+	+	+	+
Baccini 2007	+	+	+	+	+	+
Camp 2015	+	+	+	+	+	+

+	Low risk
!	Some concerns
-	High risk
D1	Randomisation process
D2	Deviations from the intended interventions
D3	Missing outcome data
D4	Measurement of the outcome
D5	Selection of the reported result

Supplementary Figure 1. Risk of bias of studies included.

Supplementary Table 2. Descriptive information of studies related to Clinical Outcomes.

Study	Study Design	Aim of the Study	Haptic Intervention Description	Stimuli Location	Study Population	Stimuli Protocol	Clinical Outcome	Results and Conclusions
Ranzani 2020	RCT	Compare robot-assisted therapy using haptic device with dose-matched conventional neurocognitive therapy for hand therapy.	ReHapticKnob Robot. A two-degrees-of-freedom high-fidelity haptic interface to train hand opening/closing and forearm rotation.	Hand and Forearm	Subacute Stroke	2 conventional neurocognitive therapy sessions 45 min robot-assisted daily sessions for over 15 days.	Motor Function (Fugl-Meyer Assessment (FMA-UE) upper extremity)	Neurocognitive robot-assisted therapy of hand function allows for a non-inferior motor recovery compared to conventional neurocognitive therapy in subacute stroke.
Baur 2018	RCT	Effects of creativity and visual display on motivation using a robotic-assisted motor therapy with a visuo-audio-haptic feedback integrated system.	ARMin arm robot generation IV. 7-degree-of-freedom exoskeleton with virtual game-like and audio-haptic environment with music allowing the selection of one out of two sound samples positioned to the left and to right.	Arm	Healthy Adults	Participants played two of four training conditions, either with (V+) or without visual display (V–). In the third round, they played a repetition of the preferred condition of the two first rounds, this time with a new V condition.	Motivation (Intrinsic Motivation Inventory (IMI))	Visuo-audio-haptic or audio-haptic environment increases motivationin robot-assisted therapy.
Hsu 2021	RCT	Differences in the sensorimotor performance of older adults' hands a single session of active Music-based interventions (MBI), and vibrotactile-enriched MBI conditions.	Vibrotactile-enriched music-based system.1) laptop computer produces music output integrated with visual information; 2) Haptic feedback component; 3) Image classifier	Fingertip	Healthy Older Adults	Single session of 30-min of vibrotactile-enriched MBI	1) Sensorimotor Performance; 2) Discriminative and Tactile Sensation.	Vibrotactile-enriched MBI improved sensorimotor performance, barognosis, and roughness differentiation compared to the active MBI group, potentially enhancing pinch performance and sensory hand functions.
Mueller 2013	RCT	To assess whether the visibility of ones' own exploratory movements impairs or enhances perceptual speed and precision of haptic stimuli with varying complexity.	Haptic Setting/Condition. With or with no observation condition (obscured vision by opaque plastic) of participants hands during manual haptic exploration.	Hand	Healthy Adults	36 consecutive stimuli with the same orientation as depicted on the display to prevent varying difficulty and exploration times due to demands of mental rotation. No time limit for exploration.	Motor Task Performance (exploration time and recognition errors)	Movement observation group made more errors compared to non-observation. Incoming visual information about own manual exploration movements increases competitive pressure for limited working memory resource.
Dobrushina 2024	RCT	Effectiveness of a body related biofeedback mimicking the natural sensations of one's own heartbeats compared previously utilized non-bodily (e.g. visual) feedback.	Woojer vibration simulator. Real-time biofeedback that mimics sensations of heartbeats. Stimulation begins when ECG R-peaks is detected, and vibration waveform mimics dynamics of pressure rise during blood ejection.	Chest (anterior middle section)	Healthy Adults	A single training session of 12 min of haptic heartbeat perception.	Interoceptive abilities (Heart rate discrimination task), Direction of attention rating (visual single-item scale)	Only haptic group showed increased interoceptive accuracy and confidence, and in a shift of attention towards the body. Real-time haptic feedback might be a superior approach to strengthen mind-body interaction.
Rowland 2024	RCT	To compare different haptic modalities (null, resistive, assistive, adaptive) for enhancing human user performance.	Virtual environment with haptic device (Geomagic Touch, 3D Systems). Utilizing the open-source library Chai3D to manage haptic rendering and record user data.	Hand	Healthy Adults	A single session of 20 minutes to complete a total of four hundred point-to-point trials using either Null, Assistive, Resistive, or Adaptive haptic feedback.	Motor Performance	Adaptive haptic feedback with faster and more effective learning in metrics of movement time, overshoot, performance index, and speed compared to other groups.
Wuang 2022	RCT	Effects of haptic perception training program on fine motor functions in adolescents with developmental coordination disorder.	Haptic Perception Training Program (HPT). Participants were instructed to touch and find the matching three-dimensional objects and choose the one identical to the sample object with designated perceptual dimensions.	Hand	Adolescents with developmental coordination disorder (DCD)	12-week training, twice a week, 30 min per session.	Motor Performance	HTP group had greater pre–post changes on fine motor integration, fine motor precision, manual dexterity, and writing. The haptic perception program showed benefits in enhancing fine motor control in adolescents with DCD.
Bortone 2020	Crossover	Compare efficacy of immersive Virtual Environments and weaRable haptic devices (VERA) compared to conventional rehabilitation of upper limb in children with Cerebral Palsy (CP) and Developmental Dyspraxia (DD).	Immersion in a virtual gaming program. It uses visual and auditory channels by means of a Head Mounted Display (HMD, Oculus Rift VK2), and through the sense of touch by means of wearable haptic devices.	Hand	Children with Cerebral Palsy and Developmental Dyspraxia	16 sessions, 2 sessions per week for 8 weeks divided in two periods by a 4-week wash-out, with a preliminary familiarization and training phase.	Motor (manual dexterity and finger coordination) and Kinesiological (movement error and smoothness) performance.	With no significant differences, both haptic and conventional group improved motor and kinesiological performance. Immersive VE and wearable haptic devices as viable alternative to conventional therapy for motor function in children with neuromotor impairments.
Childs 2018	RCT	To compare understanding of functional anatomy and Femoroacetabular impingement (FAI) pathomorphology among patients counseled with computed tomography (CT), generic hip models, and haptic 3D model.	Haptic Setting/Condition. Patients received a custom-printed 3D model of their hips) in additional to CT imaging to further review their pathoanatomy.	Hand	Adult patients diagnosed with Femoroacetabular impingement (FAI)	Patients received a custom haptic 3D model printed of their hip and received a questionary to gauge their retention of information prior to proceeding with further treatment.	Satisfaction and understanding on a variety of topics related to FAI.	Patients counseled with isolated CT imaging or haptic 3D models reported greater increases and retention of understanding compared with generic hip models alone.

Yoon 2017	Crossover	Effect of the combined haptic and skin-stretch feedbacks on the aged person's driving motor performance.	Haptic Device. A custom-designed wearable skin-stretcher and a 2D haptic joystick were developed to provide two augmented sensory feedbacks to the subject.	Hand	Healthy Elderly Adults	Session with augmented sensory feedback for guidance based on four conditions: NA (no feedback augmentation), F (force feedback augmentation by haptic joystick), C (cutaneous skin stretch feedback augmentation by skin-stretcher), FC (force feedback and cutaneous skin stretch feedback augmentations).	Control Performance	Control performance improved with combined feedback of both haptic force and skin-stretch feedback. This suggest the feasibility to improve people's task performance by synergistic effects of multiple augmented sensory feedback modalities.
Chang 2017	RCT	To investigate whether a program addressing visual–perceptual and haptic–perceptual skills can improve handwriting performance in children with handwriting deficits.	Visual-haptic perceptual training. Focused on visual perception, including visual discrimination, visual memory, visual closure, and visual searching. Additional activities required active touch without vision.	Hand	Children with Dysgraphia	Twelve sessions over 6 weeks (two per week- 45 min each) of training were administered.	Manual Skills	Along with the improved visual–perceptual skills, the experimental group showed a significant difference in far-point copy speed and handwriting accuracy.
Shire 2016	Crossover	Effects of haptic robotic system can be generalised to a standardised test of 'pen-skills', assessed using kinematic measures in children with manual coordination difficulties.	Haptic-Robotic System. It utilizes PHANTOM Omni, an impedance control haptic device which outputs a force in reaction to the user moving the input device to interact with a 3-D environment displayed on a computer screen.	Hand	Children with manual control difficulties.	Two time periods of 5-weeks were split by a 3-week gap (due to school holidays). During the intervention period, participants received haptic training and took part in one 20 minutes long session per week.	Manual Skills	The robotic haptic arm system was effective at training manual skills in children with motor difficulties. However, no generalisation of benefit was found to a manual coordination assessment battery of pen-skills.
Brown 2015	Crossover	Explore grasp and lift performance with a backdrivable terminal device placed under proportional myoelectric position control that features referred haptic feedback.	Haptic Device. A myoelectric sensor and conditioning circuit, a motorized gripper, a motorized elbow brace, a vibrotactile display, and an instrumented object.	Arm (Biceps area)	Non-amputee and trans-radial amputees	The test consisted of 144 trials sectioned into four blocks of 36 trials each. There were three conditions being tested: vibrotactile feedback, joint torque feedback, and no feedback.	Motor Performance (coordination between grip and load forces)	Minor differences in grip force, grip force coordination, and slip as a function of sensory feedback condition, though grip force at point of lift-off for the heavier object was greater for amputees with joint torque feedback.
Ang 2014	RCT	Efficacy of an Electroencephalography (EEG)-based Motor Imagery (MI) Brain-Computer Interface (BCI) coupled with a Haptic Knob (HK) robot for arm rehabilitation in stroke patients.	Haptic knob (HK) robot. Two-degree-of-freedom robotic hand interface for hand grasping and knob manipulation,	Hand	Chronic Stroke	18 sessions of intervention over 6 weeks, 3 sessions per week, 90 min per session.	Upper extremity motor function (Fugl-Meyer Assessment)	Larger motor gains in the BCI-haptic group compared to the standard therapy group at weeks 3,12, and 24. The study stands BCI-HK a effective, safe and with potential for enhancing motor recovery in chronic stroke when combined with therapist-assisted arm mobilization.
Timmermans 2014	RCT	Effectiveness and added value of the Haptic Master robot combined with task oriented arm hand training in chronic stroke patients.	Haptic Master Robot .An end-effector based robot that permits training of real-life functional tasks involving reach, grasp, as well as object transportation in a three dimensional space.	Hand	Chronic Stroke	Haptic Master Robot training was provided during 8 weeks, 4 times/week, 2× 30 min/day.	Upper extremity motor function (Fugl-Meyer); Functional Disability Level (Action Research Arm Test (ARAT); Motor Activity Log (MAL).	Haptic group with improvement after training on functional disability. However, Haptic Master robot in support of task-oriented arm training did not show additional value over the video-instructed task-oriented exercises.
Abdollahi 2014	Crossover	To test visual display and robotic technology that delivers augmented error signals during training, in participants with stroke.	Virtual Reality Robotic and Optical Operations Machine (VRROOM). 3D haptic/graphic integrated system combining display environment, robotic forces, and tracking of limb movement.	Hand	Chronic Stroke	2 weeks of training with participants receiving three 60-minute sessions per week (6 sessions per experimental phase).	Upper extremity motor function (Fugl-Meyer, Wolf Motor Function Test)	Small benefit for the EA training for upper extremity (UE) motor function. Interactive technology may improve UE motor recovery of stroke-related hemiparesis.
Cameirao 2012	RCT	To assess which features of a Virtual Reality -based Rehabilitation Gaming System (RGS) (vision-based tracking, haptics), and passive exoskeleton induce functional recovery of patients with chronic stroke.	Rehabilitation Gaming System (RGS). Vision-based tracking system, capturing movements of upper extremities by tracking colored markers positioned at specific point. For RGS-Haptics (RGS-H), RGS was coupled with a haptic interfaces (2 mechanical arms).	Arm	Chronic Stroke	35 minutes a day for 5 days a week during 4 weeks.	Upper extremity motor function. Muscle Strength, Spasticity, Synergistic Motor Patterns,	Beneficial effects of VR-based training are modulated by the use/nonuse of compensatory movement strategies and specific sensorimotor contingencies presented to the user, (visual feedback vs combined visual haptic feedback)
Zadravec 2011	RCT	To investigate the influence of haptic support algorithm (HSA) dynamics on the outcome of learning of a rather demanding two-degrees-of freedom motor task to learn wrist movements with the inferior arm.	Universal Haptic Drive (UHD) robot. It enables training of reaching movement and wrist movement by a single device. An algorithm (HSA) component added to UHD was tested in this study changing the level of haptic support according to subject's performance.	Arm	Healthy Adults	30 min training session consisted of 100 shot attempts on goal, with ball direction being randomly chosen. During training, the level of robotic support was provided to the subject as determined by one of the three dynamically different HSA.	Motor Task Performance: average error and standard deviation for upper, lower shots, before and after training.	The selection of the HSA that is appropriate for the given motor task had a significant influence on the level of acquired motor skills after the training period.
Brokaw 2011	RCT	To examine visual demonstration in a time-independent functional training (TIFT) to separate effects of action observation on motor learning from the effects of haptic guidance.	ARMin III robot. With time-independent functional training (TIFT) represents a system that matins arm movements within the proper kinematic trajectory via haptic walls at each joint.	Arm	Healthy Adults	A single training and assessment was implemented using either visual demonstration or TIFT training.	Motor Task performance: joint error, slope error, and movement variability.	TIFT subjects reduced error and interaction forces between the robot and arm, while TD subject performance did not change.

Baccini 2007	RCT	To determine effects of light touch (LT) on postural sway in older compared with younger persons.	Haptic Setting/Conditions. Light touch (LT), limited to a maximum of 1 N of applied force through right index finger. Force touch (FT), directed to exert as much pressure as necessary to stand steady through right index finger on a stable surface.	Index Finger	Healthy Adults	Subjects in semi-tandem position on firm surface while using levels of support from right hand. To control the force exerted through upper extremity, subjects put right index finger on a touch-sensitive plate. Force levels: No (NT), Light (LT), Force (FT) touch.	Postural Sway	Contact–age interaction was significant between NT and LT conditions, with older participants showing greater decrease in postural sway than younger. This suggests greater effectiveness of LT in reducing postural sway in older than in younger persons.

Supplementary Table 3. Descriptive information of studies related to Clinical Skills Training.

Study	Study Design	Aim of the Study	Haptic Intervention Description	Study Population	Area of Training	Stimuli Protocol	Clinical Outcome	Results and Conclusions
Chowriappa 2015	RCT	To compare effectiveness of Hand-on Surgical Training (HoST) technology-based urethrovesical anastomosis (UVA) versus the existing standards of training.	Hand-on Surgical Training (HoST). It simulates surgical procedures in a Virtual Reality (VR) environment, analyzing expert steps and converting them into a haptic-enabled augmented reality (AR) sequence.	Clinical Fellows and Post-Graduate Year surgery residents	Surgery	Participants were given four HoST sessions, with each session lasting for no more than 20 min.	1) UVA performance. DaVinci, Global Evaluative Assessment Skills (GEARS). 2) Cognition.National Aeronautics and Space Administration Task Load Index (NASA TLX).	HoST group obtained higher GEARS scores, and lower NASA TLX with lower temporal demand and effort. UVA Training in an AR environment improved technical skill acquisition with minimal cognitive demand.
Lim 2016	RCT	To test if mental imagery (MI) training is superior to low-fidelity (LF) haptic simulation training for epidural anesthesia skill acquisition.	Haptic Simulation Training. Low fidelity (LF) simulation using a banana to simulate the feel and resistance of inserting a needle in human tissue.	Post-Graduate Year 2 anesthesiology residents	Anesthesiology	60 minutes of LF haptic simulation training for epidural catheter placement using a banana.	Technical achievement assessed with a skills checklist. Scores (0–21, 0 the worst and 21 the best) and duration (minutes) to task completion.	No difference between the 2 groups for scores and duration to task completion. MI is not different from LF simulation training for epidural anesthesia skill acquisition.
Strom 2006	Crossover	To analyze if the addition of haptic feedback early training phase for image-guided surgical simulation (virtual upper abdomen) improves performance.	Haptic Simulator. The Procedicus abdomen is a standard personal computer (PC)-based virtual reality system for laparoscopic simulation with anatomic graphics and force feedback.	Post-Graduate Year 2-5 surgery residents	Surgery	2 hour simulator session with haptic feedback training using manipulation and diathermy (MD, 1 h) and point diathermy (PD, 1 h) as diathermy tasks.	Visual–spatial ability: BasIQ general cognitive ability test and Mental Rotation Test A (MRT-A).	The group starting with haptic feedback performed the two diathermy tasks significantly better. The haptic feedback could be important in the early training phase of skill acquisition in image-guided surgical simulator training.
Kantar 2020	RCT	To evaluate the impact of a cognitive cleft haptic surgery digital simulator on surgical skills acquisition compared to textbook.	Haptic Simulator. 3D digital animations, with text, voice over surgeon commentary, of normal and pathologic anatomy, surgical markings, and steps of fundamental cleft procedures.	Plastic Surgery Residents	Surgery	Participants were then given 15 minutes to study the markings using the haptic simulator.	Knowledge of surgical steps, procedural confidence, markings performance on a 3D stone model, and surgical performance.	Digital simulations significantly improved surgical knowledge, procedural confidence, markings performance, and surgical performance compared to textbooks.
Salvador 2024	RCT	To compare laparoscopic suturing performance in medical students when trained with different modes of real-time feedback (haptic, visual, no feedback) using a laparoscopic box trainer.	Wearable Device. Bracelet with a small vibrating motor, characterized to increase the intensity of the vibration linearly with the measured force up to its maximum intensity.	Medical Students	Surgery	8 sessions with haptic feedback.	Performance in laparoscopic suturing procedure measured by reaction force applied to the tissue.	All groups improved their tissue handling skills, being the visual group the one who achieved better performance, followed by the haptic group and lastly the control group.
Postema 2021	RCT	To investigate whether haptic exploration of an object prior to executing a laparoscopic action on it improves performance.	Haptic Training. Consisted of taking the anatomic model in hands and to explore the geometry and different structures of the model during two minutes.	Medical Students	Surgery	A 2 minutes haptic training session followed by a task session in the ForceSense box trainer.	Laparoscopic Performance	Force parameters showed lower values in in haptic group. By adding haptic sensation to the existing visual information during training of laparoscopic tasks on life-like models, tissue manipulation skills improve during training.
Hagelsteen 2019	Crossover	To validate perception and effect on performance of haptic feedback by experienced surgeons in the previously tested virtual reality simulators.	Virtual Reality Simulators with 3D imaging (LapSimVR). Instrument joysticks delivered haptic feedback interpreted as tactile sensations, or force feedback, when touching objects in virtual operating space.	Surgeons with experience in laparoscopic procedures	Surgery	The surgeons then performed a single session with five suturing attempts with haptics enabled.	Laparoscopic procedure performance.	Comparing metrics for maximum stretch damage between the groups revealed a lower score when a group performed with haptics enabled.
Alleblas 2017	Crossover	To test the effects of enhanced haptic feedback on force control, tissue consistency interpretation, and the associated surgeons' level of confidence.	Force reflecting operation instrument (FROI) training. Force measurement applied on tissue with the instrument tip and transmits information to surgeon through a resistance mechanism in the instrument handle.	Surgical Residents and Medical Students	Surgery	Participants performed three series of blinded palpation tasks, involving three different graspers: conventional grasper and the FROI grasper, which was used in the activated and deactivated states.	Surgical Performance (force control, tissue consistency interpretation, performer confidence).	Haptic feedback decreased force applied in tissue palpation, improved tissue consistency interpretation, correct and confident assessments.
Vapenstad 2017	RCT	A criterion-based training program on a VR simulator with haptic feedback was created and tested by comparing the performances of a simulator group against a control group.	LapSim VR haptic simulator. 19-inch TFT monitor, a diathermy foot pedal interface and two Xitact IHP haptic feedback instrument ports.	Medical Students	Surgery	In a single session, participants were asked to conduct a cholecystectomy using a porcine organ model.	Laparoscopic skills: depth perception, bimanual dexterity, efficiency and tissue handling of the Global Operative Assessment of Laparoscopic Skills (GOALS).	Control group achieved better video rating scores than haptic. The criterion-based training program did not transfer skills to the clinical setting.
Chao 2015	RCT	To compare the impact of virtual reality simulation training and theoretical teaching on the ability of inexperienced trainees to produce adequate virtual transvaginal ultrasound images.	Haptic transvaginal high-fidelity simulator. Ultrasound scanning procedure in a realistic, interactive, and real-time. Feedback force is applied by the simulator to the mock	Inexperienced Gynecology & Obstetrics residents	Gynecology & Obstetrics	40 minutes of virtual reality simulation training using a haptic transvaginal simulator	Gynecologic Ultrasound Skills	The quality of virtual vaginal images produced by inexperienced trainees was greater immediately after a single virtual reality simulation

			ultrasound probe manipulated by the operator.					training session than after a single theoretical teaching session.
Patel 2014	RCT	To determine if a computer-based simulation with haptic technology can help surgical trainees improve tactile discrimination using surgical instruments.	ImmersiveTouch Virtual simulator. 3D, high-resolution graphics using flat panel display along with tactile and kinesthetic feedback through two haptic devices, each used to manipulate surgical instrument simultaneously.	Medical Students	Surgery	In a single session, subjects were instructed to detect six elastothane objects of varying sizes embedded in the brain cavity model by using bipolar and suction devices. A maximum of 15 attempts for 10 minutes.	Tactile discrimination using surgical instruments.	73.3% of haptic training group found brain cavity objects compared to no virtual training (53.3%). Virtual computer-based simulators with integrated haptic technology may improve microsurgical tactile discrimination.
Thompson 2011	RCT	To evaluate the benefit of haptics in Virtual Reality (VR) laparoscopic surgery training for novices.	Simbionix LapMentor II haptic VR simulator. Simulated, computer-controlled, motorized force feedback. It has training modules for basic tasks, procedural tasks, and ability to perform full virtual procedures.	Novice laparoscopic undergraduate, medical, and graduate students	Surgery	The haptics group performed nine basic tasks to proficiency using the haptics feature of the LapMentor II, followed by a session involving laparoscopic cholecystectomy tasks.	Performance of laparoscopic cholecystectomy	The number of attempts required to reach proficiency did not differ between the haptics- and nonhaptics-training groups. In addition no difference was found in performance between groups.
Suebnukarn 2010	RCT	To examine several kinds of kinematic information about the movement provided by the system supplement knowledge of results (KR) in dental skill acquisition.	Haptic virtual reality (VR) training system. It provides force feedback to the operator's hand during probing and cutting.	Dental Students	Dental Skill Acquisition	Single task session to perform access opening on the upper molar with haptic virtual reality system. An acquisition session consisted of two days of ten trials of practice with augmented kinematic feedback.	Dental Skill Learning (overall preparation score (S))	Augmented kinematic feedback with better performance than KR in Day 1 of acquisition and retention sessions. Thus augmented kinematic feedback enhance performance in skill acquisition and retention.
Camp 2015	RCT	To evaluate the accuracy and efficiency of three patellar resection techniques: cutting guide, free hand with haptic feedback, and a novel technique utilizing four quadrants.	Freehand Resection with Haptic Training. The patella was resected in a freehand manner with an oscillating saw, and surgeon judged symmetry based on haptic feedback.	Experienced arthroplasty surgeons	Surgery	Native patellar height measured (A). Freehand resection (B) Symmetry and height assessed using haptic feedback (C). Final height measured with prosthesis in place (D). Resection repeated until the desired thickness and symmetry.	Surgical Skils (resection technique)	The most accurate methods for obtaining desired thickness were haptic feedback and novel four quadrant technique followed by the patellar cutting guide.

Section and Topic	Item #	Checklist item	Location where item is reported
**TITLE**			
Title	1	Identify the report as a systematic review.	p.1
**ABSTRACT**			
Abstract	2	See the PRISMA 2020 for Abstracts checklist.	p. 2
**INTRODUCTION**			
Rationale	3	Describe the rationale for the review in the context of existing knowledge.	p.p. 3, 4
Objectives	4	Provide an explicit statement of the objective(s) or question(s) the review addresses.	p. 4
**METHODS**			
Eligibility criteria	5	Specify the inclusion and exclusion criteria for the review and how studies were grouped for the syntheses.	p. 6
Information sources	6	Specify all databases, registers, websites, organisations, reference lists and other sources searched or consulted to identify studies. Specify the date when each source was last searched or consulted.	p. 5
Search strategy	7	Present the full search strategies for all databases, registers and websites, including any filters and limits used.	Supp Table 1
Selection process	8	Specify the methods used to decide whether a study met the inclusion criteria of the review, including how many reviewers screened each record and each report retrieved, whether they worked independently, and if applicable, details of automation tools used in the process.	p.p. 5
Data collection process	9	Specify the methods used to collect data from reports, including how many reviewers collected data from each report, whether they worked independently, any processes for obtaining or confirming data from study investigators, and if applicable, details of automation tools used in the process.	p. 5
Data items	10a	List and define all outcomes for which data were sought. Specify whether all results that were compatible with each outcome domain in each study were sought (e.g. for all measures, time points, analyses), and if not, the methods used to decide which results to collect.	p.p. 5
	10b	List and define all other variables for which data were sought (e.g. participant and intervention characteristics, funding sources). Describe any assumptions made about any missing or unclear information.	p.p. 5
Study risk of bias assessment	11	Specify the methods used to assess risk of bias in the included studies, including details of the tool(s) used, how many reviewers assessed each study and whether they worked independently, and if applicable, details of automation tools used in the process.	p.p. 5
Effect measures	12	Specify for each outcome the effect measure(s) (e.g. risk ratio, mean difference) used in the synthesis or presentation of results.	p.p. 5
Synthesis methods	13a	Describe the processes used to decide which studies were eligible for each synthesis (e.g. tabulating the study intervention characteristics and comparing against the planned groups for each synthesis (item #5)).	p. 5,6
	13b	Describe any methods required to prepare the data for presentation or synthesis, such as handling of missing summary statistics, or data conversions.	p.p. 5,6
	13c	Describe any methods used to tabulate or visually display results of individual studies and syntheses.	p.p. 5,6
	13d	Describe any methods used to synthesize results and provide a rationale for the choice(s). If meta-analysis was performed, describe the model(s), method(s) to identify the presence and extent of statistical heterogeneity, and software package(s) used.	p.p. 5,6
	13e	Describe any methods used to explore possible causes of heterogeneity among study results (e.g. subgroup analysis, meta-regression).	n/a
	13f	Describe any sensitivity analyses conducted to assess robustness of the synthesized results.	n/a
Reporting bias assessment	14	Describe any methods used to assess risk of bias due to missing results in a synthesis (arising from reporting biases).	p.p. 5
Certainty assessment	15	Describe any methods used to assess certainty (or confidence) in the body of evidence for an outcome.	n/a
**RESULTS**			
Study selection	16a	Describe the results of the search and selection process, from the number of records identified in the search to the number of studies included in the review, ideally using a flow diagram.	p.p. 6

Section and Topic	Item #	Checklist item	Location where item is reported
	16b	Cite studies that might appear to meet the inclusion criteria, but which were excluded, and explain why they were excluded.	p.p. 6; Fig. 1
Study characteristics	17	Cite each included study and present its characteristics.	Supp. Table 2
Risk of bias in studies	18	Present assessments of risk of bias for each included study.	p.p. 10<br>Supp. Fig 1
Results of individual studies	19	For all outcomes, present, for each study: (a) summary statistics for each group (where appropriate) and (b) an effect estimate and its precision (e.g. confidence/credible interval), ideally using structured tables or plots.	Supp. Table 2
Results of syntheses	20a	For each synthesis, briefly summarise the characteristics and risk of bias among contributing studies.	p. 7-9
	20b	Present results of all statistical syntheses conducted. If meta-analysis was done, present for each the summary estimate and its precision (e.g. confidence/credible interval) and measures of statistical heterogeneity. If comparing groups, describe the direction of the effect.	p.p. 7-9
	20c	Present results of all investigations of possible causes of heterogeneity among study results.	Supp. Fig 1
	20d	Present results of all sensitivity analyses conducted to assess the robustness of the synthesized results.	n/a
Reporting biases	21	Present assessments of risk of bias due to missing results (arising from reporting biases) for each synthesis assessed.	n/a
Certainty of evidence	22	Present assessments of certainty (or confidence) in the body of evidence for each outcome assessed.	n/a
**DISCUSSION**			
Discussion	23a	Provide a general interpretation of the results in the context of other evidence.	p. 10, 11
	23b	Discuss any limitations of the evidence included in the review.	p.p. 14
	23c	Discuss any limitations of the review processes used.	p.p. 14
	23d	Discuss implications of the results for practice, policy, and future research.	p.p. 15
**OTHER INFORMATION**			
Registration and protocol	24a	Provide registration information for the review, including register name and registration number, or state that the review was not registered.	p. 15
	24b	Indicate where the review protocol can be accessed, or state that a protocol was not prepared.	n/a
	24c	Describe and explain any amendments to information provided at registration or in the protocol.	n/a
Support	25	Describe sources of financial or non-financial support for the review, and the role of the funders or sponsors in the review.	
Competing interests	26	Declare any competing interests of review authors.	p. 15
Availability of data, code and other materials	27	Report which of the following are publicly available and where they can be found: template data collection forms; data extracted from included studies; data used for all analyses; analytic code; any other materials used in the review.	p. 15

From: Page MJ, McKenzie JE, Bossuyt PM, Boutron I, Hoffmann TC, Mulrow CD, et al. The PRISMA 2020 statement: an updated guideline for reporting systematic reviews. BMJ 2021;3
